# MarginView3D: A Novel Software Enabling Comprehensive Three-Dimensional Surgical Pathology Documentation for Improved Intra- and Postoperative Cancer Care

**DOI:** 10.1245/s10434-025-17695-x

**Published:** 2025-07-05

**Authors:** Michael Karasick, Justin K. Joseph, Michelle Yoon, Sabrina R. Comess, Salmaan Sayeed, Pranati Borkhetaria, Luke Stanisce, Margaret Brandwein-Weber, Mark L. Urken

**Affiliations:** 1 Department of Otolaryngology–Head and Neck Surgery, Thyroid, Head & Neck Cancer (THANC) Foundation, New York, NY USA; 2 Department of Otolaryngology–Head and Neck Surgery, Icahn School of Medicine at Mount Sinai, New York, NY USA; 3 Department of Pathology, Icahn School of Medicine at Mount Sinai, New York, NY USA; 4 Thyroid Head and Neck Cancer Foundation, New York, USA

**Keywords:** Surgical pathology report, Software innovation

## Abstract

**Background:**

Surgical pathology reports are crucial to the delivery of personalized and coordinated cancer management. However, traditional reports often lack clarity and comprehensiveness, which can lead to misinterpretation or suboptimal therapeutic planning. To address these issues, we developed MarginView3D^TM^ (MV3D), a novel surgical pathology reporting software. This software integrates real-time three-dimensional (3D) visualizations of surgical specimens, surgical defects, anatomic models, annotated radiographs, and audiovisual narrative summaries from surgeons and pathologists to enhance the care of patients with head and neck cancer.

**Methods:**

We detail the internally developed MV3D surgical pathology reporting platform created by a multidisciplinary team incorporating open-source platforms, commercial 3D scanners, and medical illustrators. This software was piloted in 26 head and neck cancer cases over 6 months.

**Results:**

The software facilitates comprehensive margin mapping and pathology reporting in a range of procedures: palatomaxillectomy (*n* = 5), parotidectomy (*n* = 5), mandibulectomy (*n* = 5), oral cavity soft tissue resection (*n* = 5), laryngectomy/laryngopharyngectomy (*n* = 4), thyroidectomy (*n* = 1), and facial cutaneous malignancy resection (*n* = 1). Notably, 24 out of 26 surgeries achieved well-documented negative final margin statuses. The median number of inadequate margins (“margins at-risk”) per case was 1.5 (range 0–6). The median number of supplemental tissue samples harvested per case was two (range 0–13).

**Conclusions:**

MV3D enhances surgical pathology documentation by consolidating precise anatomic orientation, clear margin reconciliation, and narrative summaries offered by the surgeon and pathologist into one platform to aid in the treatment of head and neck cancer. MV3D has the potential to improve margin reporting, care coordination, adjuvant treatment planning, and personalized cancer management.

**Supplementary Information:**

The online version contains supplementary material available at 10.1245/s10434-025-17695-x.

The surgical pathology report guides postoperative care and long-term management of cases of patients with cancer. Radiation and medical oncologists are informed by the reported histopathology, oncologic clearance status, and specific anatomic regions of concern when planning adjuvant therapy. Radiologists use the pathology report for guidance in interpreting postoperative surveillance imaging studies. The pathology report is of particular importance in head and neck cancer management, where resection and reconstruction drastically alter patient anatomy.^1,2^ Maximizing the clarity and comprehensiveness of pathology reports has the potential to improve postoperative care and radiologic surveillance. In contrast, ambiguous pathology reports can lead to under- or overtreatment, which can impact recovery, quality of life, and overall prognosis.^3^

The current standard pathology report can lead to misinterpretation by downstream providers. Traditionally, the report documents the results of histopathologic analysis in a bulleted, chronological format, separating gross and pathologic descriptions. This static document falls short of encompassing the extensive information gathered and discussed during surgery.^4–6^ In a survey of surgeons, pathologists, and medical and radiation oncologists assessing traditional head and neck pathology reports, 43% of respondents reported uncertainty about final margin status and expressed a need for greater clarity regarding the extent of supplemental margins harvested. Similarly, 60% of respondents questioned whether re-excised supplemental margins reflected true clear margins.^7^ This ambiguity complicates postoperative care and hampers the creation of individualized adjuvant treatment strategies, particularly if care is fragmented across multiple institutions and practice settings.

Despite significant advances in technology, the processes of pathologic assessment and documentation have remained stagnant. Over the past 5 years, we have developed an intraoperative, interdisciplinary workflow that allows for visualization of anatomically based margin reporting. The core of this novel workflow utilizes intraoperative three-dimensional (3D) scans and a series of strategic “timeouts” to document and communicate the precise locations of frozen section margins on the surgical specimen and any required supplemental margins on the ablative defect.^8–10^ The result is a final surgical pathology report that integrates annotated 3D scans, preoperative radiographs, and succinct narrative summaries by the surgeon and pathologist.^7,11^

Our efforts laid the groundwork for designing a novel surgical pathology reporting system, MarginView3D^TM^ (MV3D). We developed this software as an interdisciplinary and Health Insurance Portability and Accountability Act of 1996 (HIPAA)-compliant system. MV3D goes beyond a static document of histopathological data reporting and provides a visual, interactive, and dynamic representation of the actions taken and information obtained during oncologic surgery. The software produces a dynamic document that clearly identifies the final oncologic status and areas of clinical interest (i.e., at-risk margins) with greater visual detail, facilitating the planning of targeted adjuvant therapy. Additionally, the transfer of anatomic information from the 3D specimen and defect scans to annotated preoperative radiographs bridges the information gap from intraoperative details to radiographic images. These annotated radiographs serve as the visual currency used by clinicians throughout a patient’s postoperative cancer journey. Ultimately, MV3D aims to improve oncological documentation and physician workflow by clarifying ambiguous anatomic relationships. The point-to-point correlation between intraoperative margins at risk and harvested supplemental margins allows the pathologist to report a patient’s final oncologic status more confidently.

## Methods

This study was conducted following approval from the Mount Sinai Health Systems Institutional Review Board (STUDY-23-01174). MV3D was developed by a multidisciplinary team comprised of software engineers, surgeons, pathologists, medical illustrators, and clinical research staff. It was created with guiding principles of enhanced cancer treatment, patient safety, accessibility, and clarity. The system is completely written using open-source software, fostering global medical and technological community participation in its continuous improvement. The program is built on the Three.js® platform, an open-source JavaScript library that displays animated 3D graphics within a web browser. MV3D leverages this technology to provide detailed, interactive 3D visualizations of surgical specimens and defects, as well as digitally constructed 3D anatomical head and neck models of the affected sites.

**Fig. 1:**
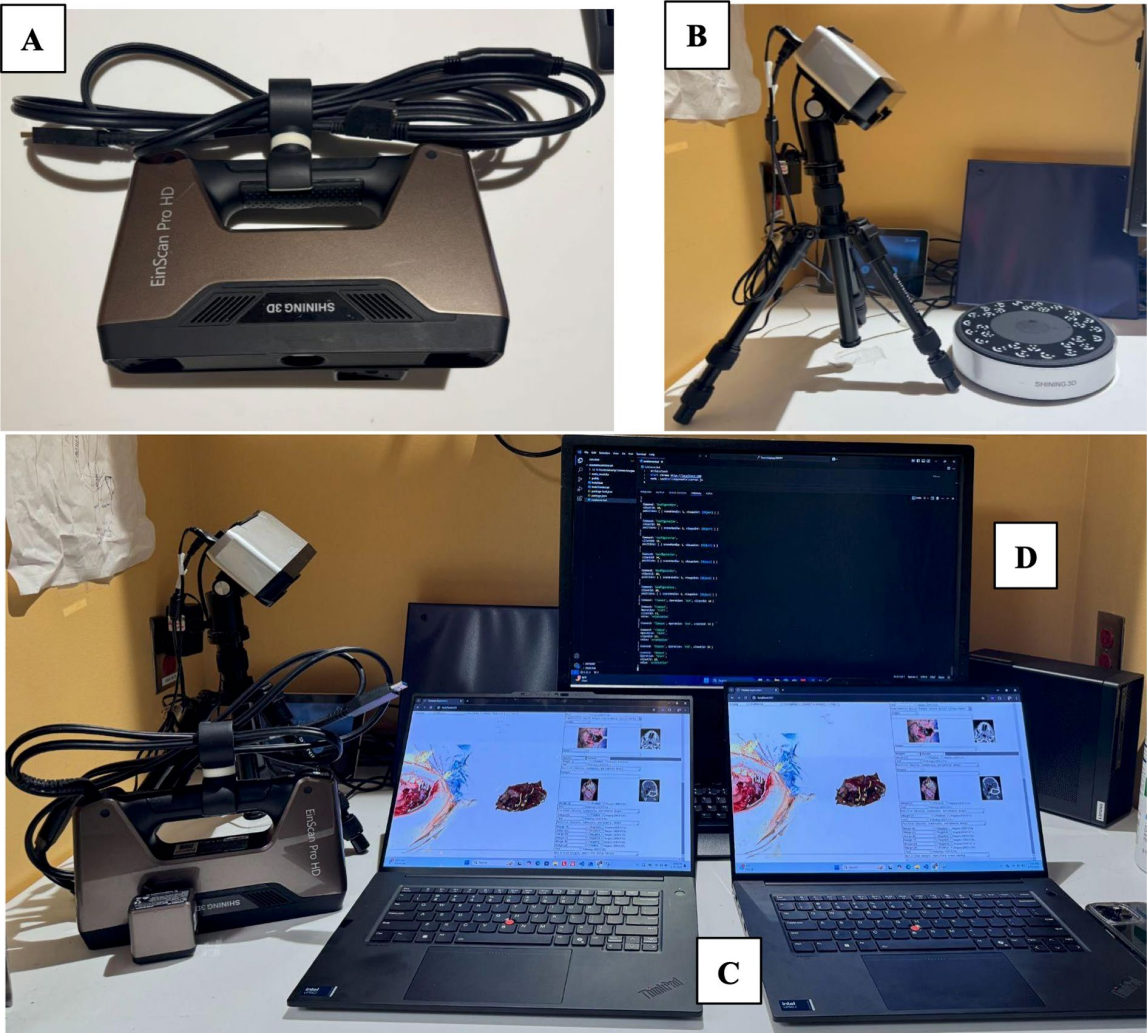
The equipment used for 3D scanning in our study comprises two commercial off-the-shelf (COTS) 3D scanners, the handheld EinscanProHD® **A** and the tabletop Einscan-SP® **B**, both from Shining 3D (Hangzhou, China). Two Lenovo touchscreen laptops **C** and a Lenovo server **D** are used in conjunction with the scanners

For defect and specimen scanning, we used two different commercial off-the-shelf (COTS) 3D scanners: a handheld EinscanProHD and a tabletop Einscan-SP®, both manufactured by Shining 3D (Hangzhou, China). These scanners are selected for their high precision (up to 0.04 mm fidelity) in capturing detailed 3D images, essential for accurate defect and specimen analysis (Fig. 1). Scans are imported into MV3D as standard Wavefront. OBJ files (including texture and material).

The operational setup also includes two COTS Windows-based Lenovo® touchscreen laptops and a COTS Lenovo® server. The touchscreen capability of the laptops is essential to facilitate direct annotation of 3D scans. One laptop is placed in the operating room for the surgeon, and the other is placed in the frozen section laboratory for the pathologist. The COTS server transforms the margin descriptions into an evergreen document, which allows real-time edits and changes from different users in the operating room and the pathology laboratory. MV3D permits surgeons and pathologists to visualize and manipulate surgical margins collaboratively during and after surgery.

A comprehensive 3D anatomical library was developed by a medical illustrator in collaboration with surgeons to produce anatomically accurate models of the head and neck using ZBrush®. The segmentation of bony anatomy was rendered from computed tomography (CT) and magnetic resonance imaging (MRI) scans, while soft tissue representations were digitally illustrated and adjusted under the supervision of senior surgeons. The library encompasses 55 distinct anatomical structures, offering customizable features to match the specific anatomy of individual patients and surgical viewpoints. Models can be constructed to show a variety of anatomic parts, including the total and partial mandible, various tongue retractions, and buccal mucosa retraction.

A pilot validation study using MV3D was conducted from August 2024 to January 2025. MV3D was introduced into the clinical environment, where it was successfully used in 26 consecutive head and neck cancer resections performed at Mount Sinai West Medical Center in New York City, NY. These cases all required frozen section analysis in accordance with the standard of care and our traditional workflow. The parameters collected for each case in this study include: the type of ablative procedure; the number of margins at risk; the annotation of specimen scans, defect scans, and anatomic models; and the number of supplemental margins harvested.

## Results

MV3D was utilized across a diverse array of procedures: palatomaxillectomy (*n* = 5), parotidectomy (*n* = 5), mandibulectomy (*n* = 5), oral cavity soft tissue resection (*n* = 5), laryngectomy/laryngopharyngectomy (*n* = 4), thyroidectomy (*n* = 1), and facial cutaneous malignancy resection (*n* = 1) (Table 1). The median age was 70 years old (range 42–89 years). Of the 26 cases, 15 (58%) were male, 19 (73%) were White, and 7 (27%) were Black. All cases included specimen scanning, and 24 cases were further accompanied by a defect scan. The absence of defect scans (one mandibulectomy [case 17] and one glossectomy [Case 18]) was due to anatomical constraints, i.e., trismus, that precluded clear 3D rendering of the deeper regions of the cavity. These cases were then supplemented with 3D anatomical models of the surgical site in lieu of the defect scan. In total, 18 cases were enhanced by incorporating the corresponding 3D anatomical models within MV3D.

**Table 1:** Case characteristics of MarginView3D pilot test

Case	Age (years) and sex; race	Procedure	Specimen scan (Y/N)	Defect scan (Y/N)	Anatomical model (Y/N)	Margins at risk	Supplements	Final margin status
1	76M; White	Mandibulectomy	Y	Y	N	0	0	Negative
2	42F; Black	Parotid	Y	Y	N	0	0	Negative
3	67F; White	Mandibulectomy	Y	Y	Y	0	0	Negative
4	61M; White	Parotid	Y	Y	N	3	3	Negative
5	69F; Black	Parotid	Y	Y	N	0	0	Negative
6	71M; White	Parotid	Y	Y	N	0	0	Negative
7	89F; White	Palatomaxillectomy	Y	Y	Y	2	2	Negative
8	78M; White	Laryngopharyngectomy	Y	Y	Y	4	4	Negative
9	67M; Black	Parotid	Y	Y	N	0	0	Negative
10	71M; White	Palatomaxillectomy	Y	Y	Y	2	1	Negative
11	82F; White	Oral cavity soft tissue	Y	Y	Y	2	1	Negative
12	59F; Black	Laryngopharyngectomy	Y	Y	Y	4	5	Positive^*^
13	81M; White	Mandibulectomy	Y	Y	Y	3	3	Negative
14	69F; White	Oral cavity soft tissue	Y	Y	Y	6	6	Negative
15	69M; White	Laryngopharyngectomy	Y	Y	Y	0	1	Negative
16	63M; White	Palatomaxillectomy	Y	Y	Y	3	6	Negative
17	70M; White	Mandibulectomy	Y	N	Y	1	2	Negative
18	67F; Black	Oral cavity soft tissue	Y	N	Y	0	4	Negative
19	79M; White	Laryngopharyngectomy	Y	Y	N	0	0	Negative
20	88F; Black	Palatomaxillectomy	Y	Y	Y	1	2	Negative
21	54M; White	Thyroidectomy	Y	Y	N	0	0	Negative
22	82M; White	Palatomaxillectomy	Y	Y	Y	3	13	Negative
23	68M; White	Oral cavity soft tissue	Y	Y	Y	6	6	Negative
24	73M; White	Oral cavity soft tissue	Y	Y	Y	3	3	Negative
25	69F; Black	Facial cutaneous malignancy	Y	Y	Y	0	0	Negative
26	71F; White	Mandibulectomy	Y	Y	Y	4	5	Positive^**^

*F* female, *M* male, *N* no, *Y* yes

^*^Case 12 maintained a positive final margin status, as the surgery was terminated early due to extensive lymphovascular invasion

^**^Case 26 maintained a positive final margin status, as a positive bony margin was identified after surgery on permanent pathology

Regarding surgical margins, 24 out of 26 cases achieved a negative final margin status (Table 1). One case maintained a positive final margin status, as the resection was terminated due to extensive lymphovascular invasion precluding surgical intervention. A second case had a positive bone margin that was identified on permanent pathology. The median number of margins at risk per case was 1.5 (range 0–6), and the median number of supplemental tissue samples harvested per case was two (range 0–13). Importing scans into MV3D took approximately 10 s, with margin annotation taking 3–5 min and recording narrative summaries taking 2–4 min. Total workflow time ranged from 5–9 min.

**Fig. 2:**
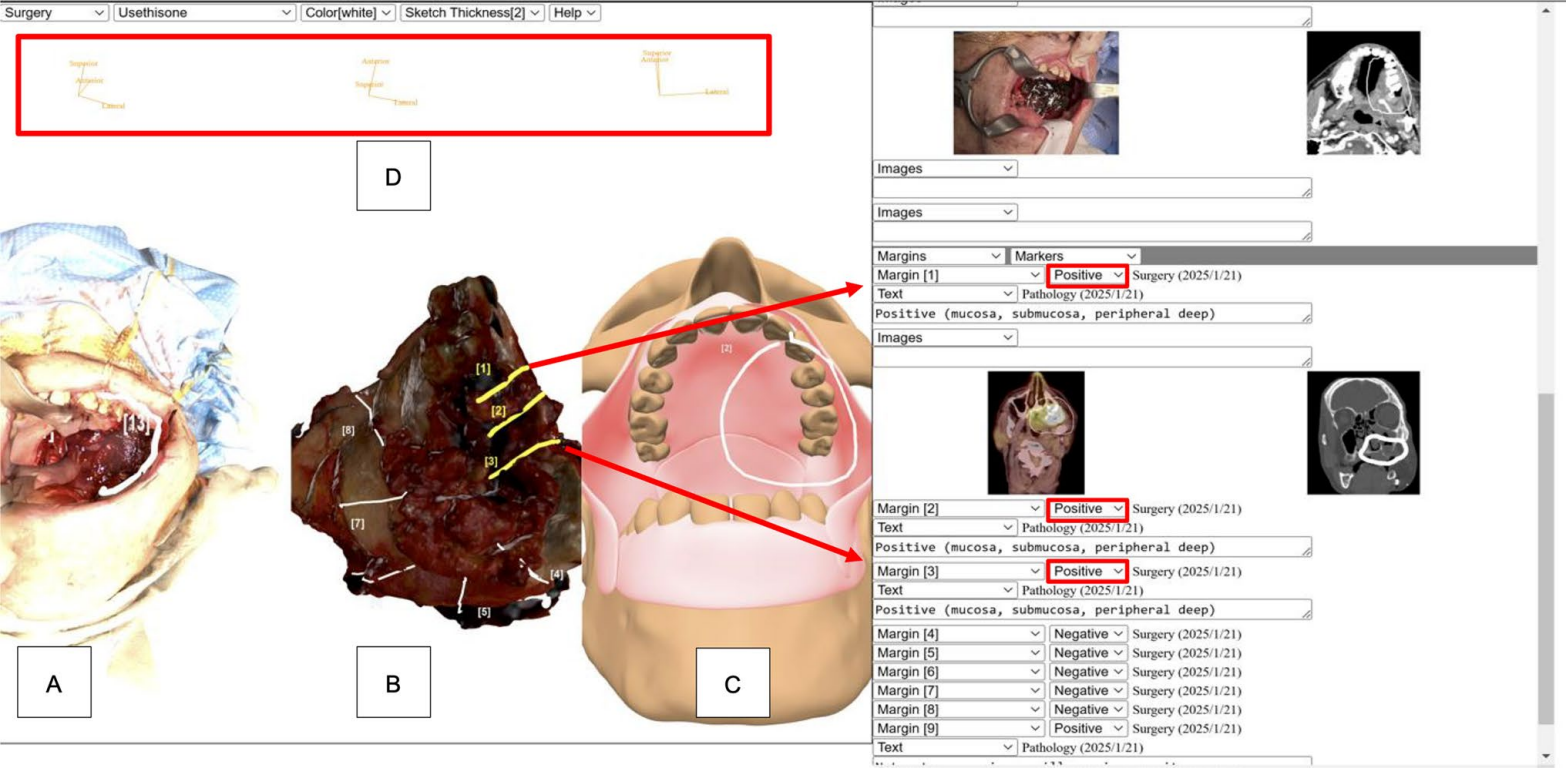
The MarginView3D^TM^ application is shown with its component features. The “Complete Resection Margin Data” view is demonstrated, including a detailed, annotated 3D visualization of the surgical defect **A**, specimen **B**, and 3D anatomical model **C**. All visuals are oriented on a common coordinate system **D**. Each margin is numbered and color-coded on the basis of intraoperative and pathologic assessments (yellow = positive margins; white = negative margins). The surgeon annotates the defect to reflect the location of the supplemental margin(s) in response to positive margins reported by the pathologist. The 3D anatomical model is used to highlight the extent of resection. Annotated radiographic studies **E** enhance understanding of the precise location of each margin and/or resection area, facilitating more precise adjuvant treatment planning

We illustrate Case 22, an 82-year-old male patient with palatomaxillary cancer undergoing an infrastructure maxillectomy, documented using MV3D (Fig. 2). Both the specimen and defect were scanned and imported into MV3D. The 3D anatomical model created by the surgeon included the following components: soft and hard palate, maxilla, mandible, upper and lower gingiva, buccal mucosa (retracted), floor of mouth, and tongue. The pathologist analyzed and annotated the specimen, identifying nine margins sampled, three of which were deemed to be at risk during frozen section margin mapping. In response, the surgeon took a total of 13 supplemental margins. The 13th supplement was specifically annotated in response to the three at-risk margins on the specimen. The 3D model was annotated by the surgeon to highlight the area of resection. All three elements—the defect, specimen, and anatomic model—were orientated anatomically on the *x*-, *y*-, and *z*-axes, labeled superior, anterior, and lateral, respectively. Preoperative radiographs were also included in this pathology report, annotated by the surgeon to delineate the extent of surgery as well as the location where supplemental margins were taken to address margins at risk.

## Discussion

The results of this pilot study demonstrate the effective and practical application of our novel surgical pathology-focused software in head and neck cancer surgery. The variety of procedures demonstrates the versatility and wide applicability of the software. The case description serves to highlight the unique features of MV3D by clearly documenting and illustrating intraoperative actions taken to achieve oncological clearance. MV3D improves on numerous shortcomings of traditional surgical pathology reporting, specifically related to intraoperative workflow, anatomic orientation, margin reconciliation, and downstream utilization.

### Improved Surgical–Pathological Workflow

3D visualization has advanced surgical pathology reporting and transformed intraoperative discussions.^7,8,12–16^ The initial iterations to this workflow included implementing 3D scans of the surgical specimen, which the pathologist uses to document how the specimen is analyzed as well as the results of that analysis.^8^ Subsequent enhancements introduced the use of 3D surgical defect scans, enabling surgeons to document the location and breadth of supplemental margins.^9^ The inclusion of the “Frozen Section Timeout” during surgery was implemented to enhance intraoperative communication and to facilitate the clear presentation of final intraoperative margin status based on primary and supplemental margin reconciliation.^10^ This timeout, plus the results of additional sections taken, enables the pathologist to more confidently generate a “final margin status” statement in the permanent pathology report.

Several limitations in our prior published workflow led to the development of MV3D. They were (1) a lack of streamlined workflow due to the involvement of multiple third-party applications; (2) a lack of a standardized surgical pathology reporting process; (3) an inability to utilize 3D defect scans for deep intraoral and pharyngeal resections; and (4) the transferability of the final product to downstream providers.^10^ Previous efforts to integrate 3D scanning technologies involved a variety of tools, such as Microsoft Paint 3D®, Procreate®, Blender®, and Microsoft Excel®. These tools, while individually powerful, led to a cumbersome, multi-software approach that failed to provide a seamless transfer of data and lacked the precision required for accurate surgical reporting. MV3D streamlined this process through a single unified platform with standardized results.

### Improved Margin Clarity and Anatomical Orientation with MV3D

Positive oncologic margins portend worse patient outcomes.^17^ Obtaining clear margins can potentially obviate the need for additional surgery and/or adjuvant multimodal therapy, both of which can negatively impact patient function and quality of life.^18–20^ Intraoperative frozen section analysis, the gold standard for margin assessment, guides surgical decisions for oncologic clearance.^16^ The specimen-driven approach to margin analysis, in comparison to the defect-driven method, is associated with better local control rates, but it is not without shortcomings.^21–25^ This method requires surgeons to accurately correlate at-risk (positive or < 5 mm) margins on the specimen with precise iterations of supplemental margins from the defect bed.^26,27^ Specimen-driven frozen section analysis is hampered by disassociation of where the margins on the specimen match up with the corresponding location on the defect. Previous literature has demonstrated that, in one-third of cases, supplemental margins are off target by up to 1 cm.^28^ This process can be imprecise, leading to ambiguity in documenting the extent and location of supplemental margin resection. Ambiguity in naming harvested supplemental margins limits a pathologist’s confidence in issuing a final margin status. Similarly, it is up to the reader of a pathology report to correlate supplemental margins with specimen margins at risk and determine whether the surgical team achieved oncologic clearance.

Significant discrepancies between frozen section and final pathology results are often attributed to errors in intraoperative interpretation hindered by differing perspectives. Surgeons operate with reference to the anatomical defect, whereas pathologists assess the specimen in isolation.^29–31^ Additionally, the process of frozen section erases discernible visual landmarks on the specimen, complicating the accurate identification of at-risk margin locations within the defect.

**Fig. 3:**
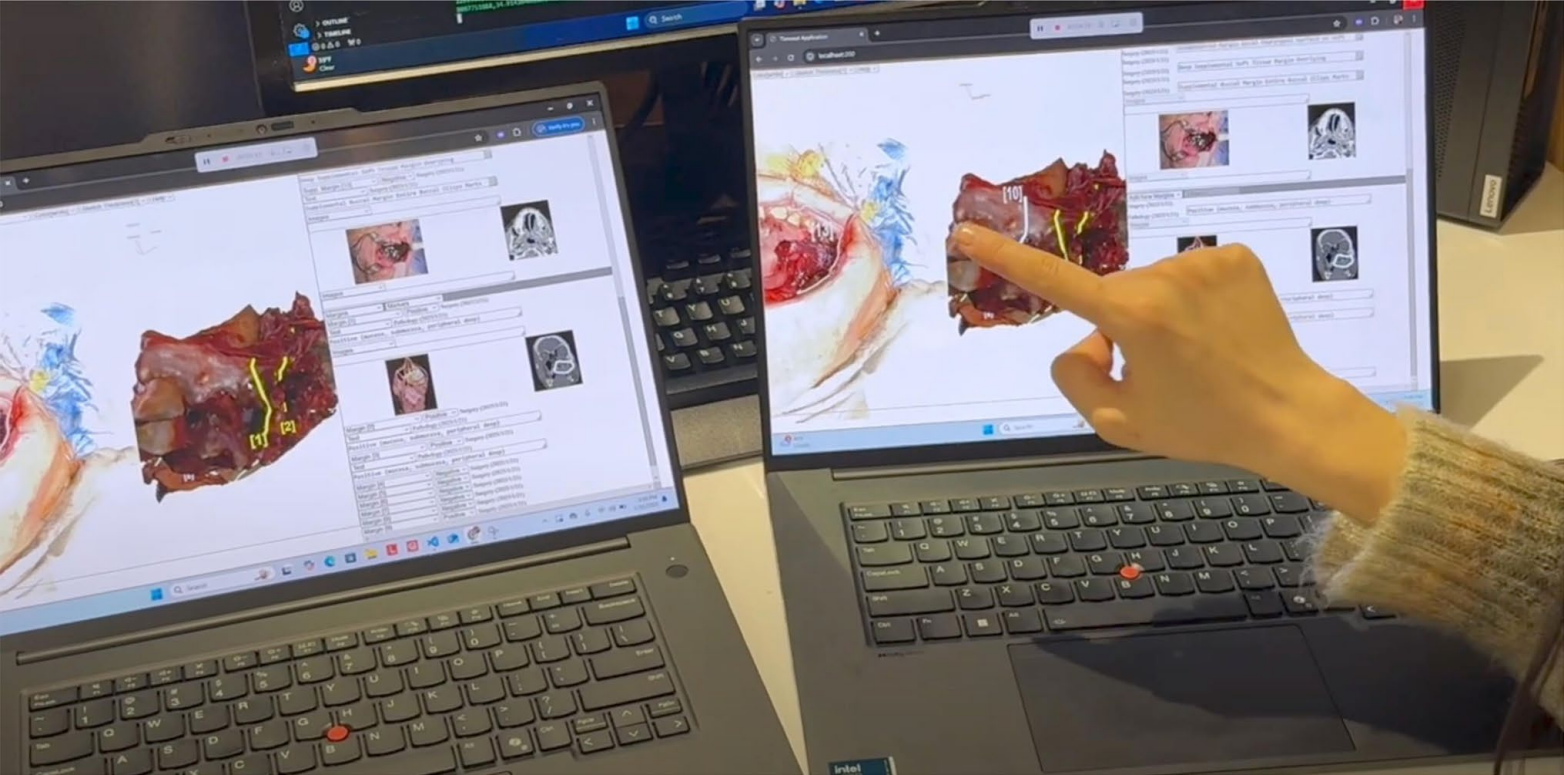
Intraoperative utilization of MarginView3D^TM^: MarginView3D^TM^ (MV3D) facilitates the importation of 3D scans for the specimen and defect, along with creating patient-specific 3D anatomical models. Orientation is performed immediately after the 3D scans are completed and before the frozen section dissection begins. Each axis is labeled using standard anatomic orientation terms to represent the anatomic position of the 3D visual. Annotations on the specimen highlight margins taken, and annotations of the defect and model delineate the area of resection and supplemental margins. MV3D supports bi-directional communication during surgery through its display in both the operating room and the frozen section laboratory. Key timeouts include an “Orientation Timeout” to confirm the alignment of the specimen with the defect, a “Frozen Section Timeout” for discussing margin status and guiding the harvest of supplemental margins, and a “Reconciliation Timeout” at the conclusion of the case to confirm and communicate surgical actions taken to achieve oncological clearance of at-risk margins (https://youtu.be/dFLlOEVq7tg?feature=shared)

MV3D facilitates a comprehensive and coherent exchange between the surgeon and pathologist for anatomic orientation, enhanced by real-time visualization of the 3D scans of both the specimen and defect. The process of achieving that anatomic correlation is documented in an “Orientation Timeout.” Before frozen section analysis, the specimen and defect model are oriented collaboratively (co-registered) according to a common coordinate system, improving precise visualization of how the specimen fits into the patient’s anatomy (Fig. 3).

Furthermore, MV3D enables real-time communication and documentation of each margin’s location and status. Following frozen section analysis, the pathologist annotates the precise location and status of each margin on the 3D specimen. After confirming proper anatomic orientation, MV3D is projected onto the operating room monitor. Simultaneous viewing and manipulation of the specimen and defect scans facilitate collaborative discussion to relay pathology results, referred to as the “Frozen Section Timeout.” During this timeout, the pathology team highlights the precise locations of at-risk margins on the specimen, promoting collaborative determination of where supplemental margins should be harvested from the defect. MV3D enhances the mapping of the specimen onto the surgical defect by enabling synchronized visualization of the two scans. This feature allows the user to move the specimen and defect in tandem, in opposition, or individually to further emphasize their interrelatedness.

**Fig. 4:**
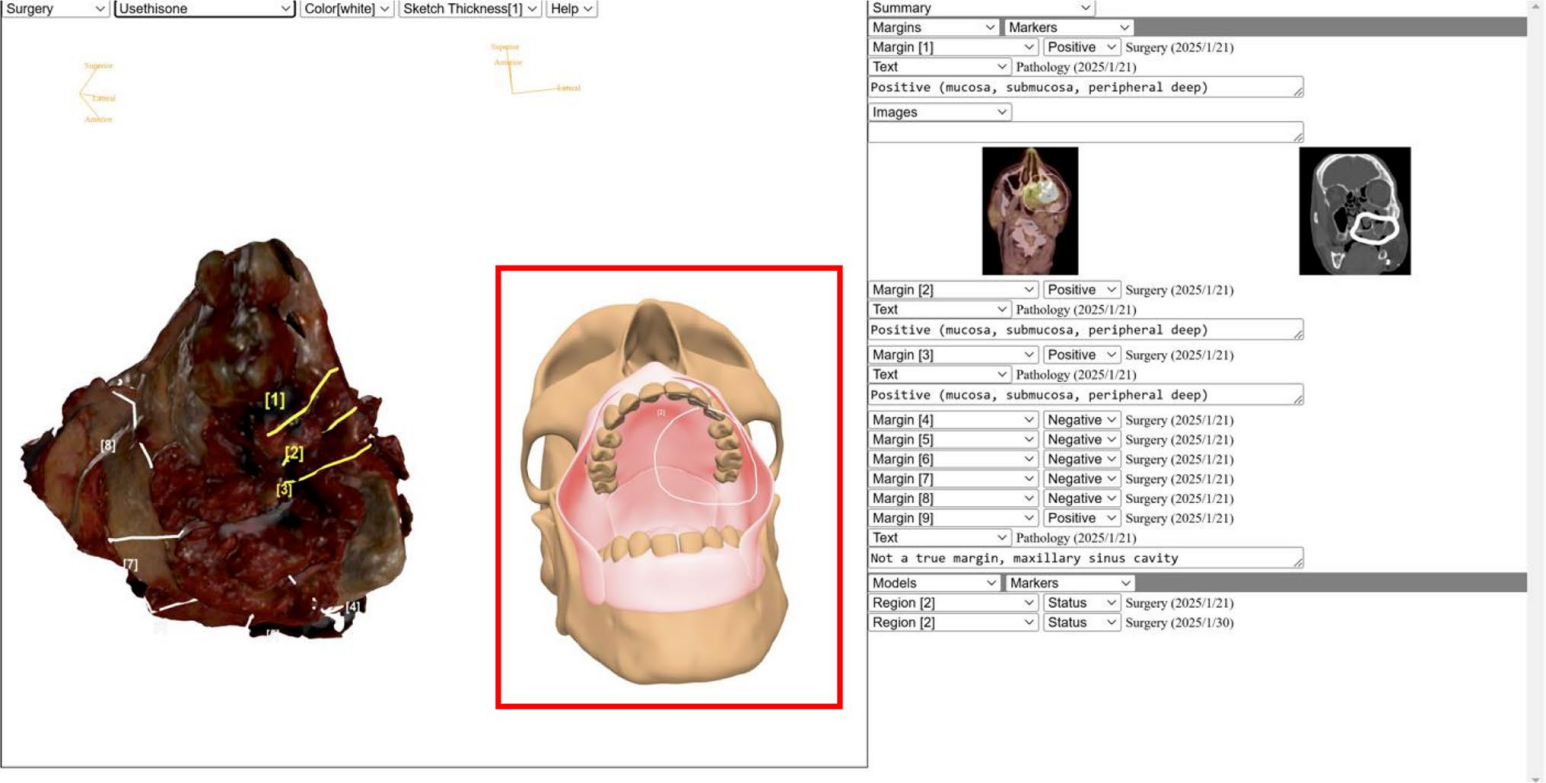
Video demonstration of the Modular Anatomical Model Library. MarginView3D^TM^ has a novel comprehensive modular 3D head and neck anatomical library. This feature allows surgeons to select, annotate, and manipulate specific anatomical features to accompany and enhance the display of the 3D defect model (https://youtu.be/6yyeExjUYLo?feature=shared)

For regions where defect scanning is not feasible using current handheld scanning technology, MV3D includes a novel library of 3D anatomical models (Fig. 4). Limitations in defect scanning include anatomical constraints (e.g., posterior oral cavity, oropharynx, hypopharynx, and sinonasal tract, as well as patients with trismus). Likewise, certain defect scans may benefit from supplemental representation of the pertinent anatomy on a 3D model that can be built to the specific needs of the patient undergoing surgery. Surgeons can fully customize these models to create a realistic representation of a patient’s anatomy, clearly visualizing the area of interest and annotating both the area of resection and the locations of supplemental margins. Using MV3D, the annotated 3D model is then displayed alongside the specimen and/or defect scans.

Postoperatively, the surgeon can precisely annotate the 3D defect scan and/or anatomic models to indicate the location and extent of supplemental margins taken in response to identified at-risk margins on the specimen. The integration of annotated, dynamic 3D optical scans into the frozen section process through a visually collaborative system significantly improves real-time communication and clarity, leading to a more precise harvest of supplemental margins.

### Addressing Downstream Communication: Bridging the Gap between Surgical Intervention and Postoperative Care

Oncologists and radiologists navigate complex data, including operative notes, pathology reports, and imaging studies to understand surgical interventions and margin status. When ambiguities are identified in the final pathology report that necessitate further clarification, downstream providers often engage in time-consuming, informal discussions with the surgical and pathology teams that are conducted weeks after surgery. Inadequate documentation or imprecise recall may prompt adjuvant treatments that impart undue toxicity to the patient and unnecessary expense to the healthcare system.^32–34^ Additionally, current surgical pathology reporting cannot convey nuanced anatomical changes that result from surgery. Radiologists analyzing postoperative imaging face the challenge of reconciling preoperative studies with drastically altered postsurgical anatomy. This can challenge their ability to identify clinically significant areas that demonstrate residual disease and/or require close monitoring. Radiation oncologists similarly rely on both pre- and postoperative imaging to delineate treatment volumes and boost dosing to specific areas of concern.^35^

MV3D allows surgeons to import radiographic studies, which can be annotated to highlight the extent of resection and the specific location(s) of supplemental margins harvested, pinpointing areas potentially harboring residual disease. Specific images may be selected that best convey the extent of surgical interventions, margins remaining at risk, and other regions of concern. The goal of achieving an improved understanding of the surgical impact on a patient’s anatomy is accomplished by the creation of a visual link between the ablative surgery and annotated preoperative imaging.

An additional feature of MV3D is the ability to capture audiovisual narratives from both surgeons and pathologists, documenting the details of the surgical case and pathologic evaluation. This enables surgeons to describe and visually demonstrate surgical interventions on 3D models, defects, and radiographs with more detail and anatomic understanding than a traditional operative report. Pathologists can further complement this by recording descriptions of the specimen analysis, including permanent pathologic analysis, and enhancing understanding through visualizations. Downstream providers may quickly and effectively reference these key details of the surgical pathology report communicated by the surgeon and pathologist directly involved with the case. Especially in more complex resections, these narratives provide a much greater degree of clarity than can be easily communicated through conventional written forms of documentation.

**Fig. 5:**
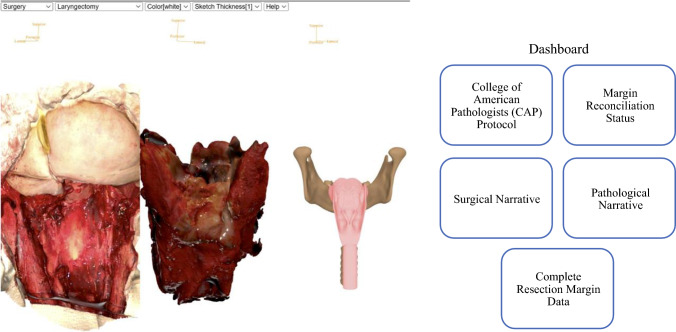
Video demonstration of the Pathology Dashboard for options for review of pathology data contained within MarginView3D^TM^. This video demonstrates how surgeons and pathologists create narrative summaries of the procedure through MarginView3D^TM^ (MV3D), as well as the unique views clinicians can access through the Pathology Dashboard. There are five views available: a PDF of the Pathology Report and the College of American Pathologists (CAP) Protocol; Margin Reconciliation Status, a detailed view highlighting margins at risk and their corresponding supplemental margins taken to clear them; Surgical Narrative; Pathological Narrative; and Complete Resection Margin Data, a summary view of all margins and supplements taken. These views guide the clinician through MV3D and describe the extent of surgery and pathological evaluation, allowing downstream providers a succinct, clear, and visual understanding of the results of a patient’s surgical treatment (https://youtu.be/Fssqfce97iY?feature=shared)

When attempting to understand a patient’s postoperative pathology, MV3D allows clinicians to select the information that they would like to review in the final pathology report by choosing from five different options located on the “Pathology Dashboard” (Fig. 5). The first option is the PDF of the standard “Pathology Report and the College of American Pathologists (CAP) protocol,” which details the final diagnosis, tumor characteristics, pathologic stage classification, and gross specimen descriptions. The second option is the “Margin Reconciliation Status,” which lists only the margins at risk and the associated specific supplemental margins that were harvested to clear and address them. In addition, this view includes a display of annotated 3D scans of the specimen and the defect and/or model that highlights the location and breadth of specific supplemental margins that were taken for each at-risk margin. The third option is the “Surgical Narrative,” which is a recorded audiovisual presentation by the surgeon that describes the details of the surgical actions taken to achieve oncologic clearance. This narrative is recorded at the completion of the surgery when the information is fresh in the surgeon’s mind. The fourth option is the “Pathological Narrative,” which is recorded by the pathologist several days postoperatively as an audiovisual presentation includes details of the final pathologic analysis, and in particular highlights any discrepancies from the information reported by the surgeon. The fifth option is the “Complete Resection Margin Data,” which enumerates all margins taken from the specimen and supplemental margins taken from the defect, providing descriptions, margin statuses, corresponding radiographic images, and annotated 3D scans of the specimen, defect, and/or model.

MV3D streamlines the creation of comprehensive pathology reports and supports real-time collaboration and documentation. All users within the hospital computer network may access and work on pathology reports concurrently, either from the operating room or frozen section laboratory. Such automation markedly reduces the time and effort required for detailed and accurate report creation. Moreover, this server-based system enhances information transfer to downstream providers, allowing streamlined access to crucial data. Our current protocol permits any healthcare provider on the patient’s care team within the institution to access a patient’s 3D pathology report. Upon completion of the surgical pathology report, including the audiovisual narratives, the final product is shared as a read-only version to all members of a patient’s care team. This integration improves interaction with surgical and pathological data, addressing delays in postoperative discussions and significantly improving the reliability of information for subsequent treatment planning.

### Limitations

Although MV3D is engineered with a user-friendly interface, new users may experience a learning curve to fully deploy its features. To overcome this challenge, we have integrated a *New User Guide* within the application designed to assist any uninitiated clinician in creating, as well as reviewing, surgical pathology reports effectively. As with any new software, greater experience with its use and the altered workflow that it supports will lead to greater efficiency. Currently, the adoption of MV3D requires the purchase of commercially available hardware and commitment to adoption by both surgeons and pathologists.

Whether the use of 3D scanning and MV3D will improve margin clearance and patient outcomes remains to be determined. Our current goal was to address potential reporting ambiguities that leave the final margin status in doubt. This investigation sets the stage to explore potential downstream effects of MV3D, such as impacting the design of postoperative radiation fields and assisting in radiographic detection of recurrences. Additional investigations assessing clinical outcomes compared with matched controls with larger cohorts and longer follow-ups are necessary.

## Conclusions

MV3D is the first independently created system aiming to standardize surgical pathology reporting. The results of this single-institution pilot study demonstrate the effective and versatile application of the software program in head and neck oncologic surgery. The features of MV3D help streamline intraoperative workflows, enhance shared anatomic understanding, and eliminate margin ambiguity. By transforming the traditional pathology report into a tool that clarifies surgical actions in real time, MV3D aims to bridge the gap between operative and postoperative care by significantly improving the dissemination of comprehensive and concise information. Future studies will explore the software’s clinical implications and applicability to other cancer surgeries across institutions.

## Supplementary Information

Below is the link to the electronic supplementary material.

Supplementary file1 (MP4 9533 kb)

Supplementary file2 (MP4 3142 kb)

Supplementary file3 (MP4 3491 kb)
